# Risk Factors for Wound Complications in Vulvar Cancer Surgery and Indications for Reconstructive Surgery

**DOI:** 10.3390/cancers17111749

**Published:** 2025-05-23

**Authors:** Justin J. E. Delahaije, Ephrahim E. Jerry, Saskia Houterman, Ashley van Woerkom, Doremieke van Loosdregt, Dorry Boll, Brigitte F. M. Slangen, Ruud L. M. Bekkers, Peggy J. De Vos van Steenwijk, Joanne A. de Hullu, Annemijn J. W. M. Aarts, Emiel L. W. G. van Haren, Edith M. G. van Esch

**Affiliations:** 1 Department of Gynecology, Catharina Hospital Eindhoven, 5623 EJ Eindhoven, The Netherlandsephrahim.jerry@catharinaziekenhuis.nl (E.E.J.); ashleyvanwoerkom@hotmail.com (A.v.W.); doremieke.v.loosdregt@catharinaziekenhuis.nl (D.v.L.); dorry.boll@catharinaziekenhuis.nl (D.B.); ruud.bekkers@catharinaziekenhuis.nl (R.L.M.B.); 2 Department of Plastic and Reconstructive Surgery, Catharina Hospital Eindhoven, 5623 EJ Eindhoven, The Netherlands; emiel.v.haren@catharinaziekenhuis.nl; 3 Department of Research, Catharina Hospital, 5623 EJ Eindhoven, The Netherlands; 4 Department of Gynecology, Maastricht University Medical Center, 6229 ET Maastricht, The Netherlands; brigitte.slangen@mumc.nl (B.F.M.S.); peggy.de.vosvansteenwijk@mumc.nl (P.J.D.V.v.S.); 5 GROW—School for Oncology and Developmental Biology, Maastricht University, 6229 ER Maastricht, The Netherlands; 6 Department of Gynecology, Radboud University Medical Center, 6525 GA Nijmegen, The Netherlands; joanne.dehullu@radboudumc.nl; 7 Department of Obstetrics and Gynecology, Cancer Center Amsterdam, Amsterdam University Medical Centers, Vrije Universiteit Medisch Centrum, 1081 HV Amsterdam, The Netherlands; j.w.m.aarts@amsterdamumc.nl

**Keywords:** vulvar cancer, reconstructive surgery, risk factors, wound complications

## Abstract

**Objective**: Vulvar cancer surgery is associated with high postoperative wound complication rates. Reconstructive surgery (RS) in vulvar cancer is generally reserved for surgery of extensive tumors or local recurrences. The primary aim of the study is to determine the incidence and risk factors for wound complications after vulvar cancer surgery. As a secondary aim, we compare the effects of primary closure (PC) versus reconstructive surgery on wound complications. **Methods**: In a retrospective cohort study in four gynecologic oncology centers in the Netherlands, patients undergoing surgical treatment (2018–2022) for vulvar cancer were included. Wound complications after PC and RS and risk factors associated with complications were analyzed by using logistic regression adjusting for confounds. **Results**: We included 394 women, 318 with PC and 76 with RS. The incidence of wound complications was 46.7%, with 42.4% of wound breakdowns comprising the majority of complications. The use of RS was associated with an increased risk of wound complications. Larger tumor size, proximity to the urethra, resection of the urethra during surgery, and perineal tumor location were additional risk factors for wound complications. However, after multivariate analyses, RS remained the only significant risk factor (OR 1.2; 95% CI 1.1–1.2). **Conclusions**: Risk factors for wound complications after vulvar cancer surgery include larger tumor size, proximity to the urethra, resection of the urethra during surgery, and perineal tumor location. RS is also associated with an increased risk of wound complications, probably related to case selection.

## Simple Summary

Wound complications are a frequent and significant challenge in vulvar cancer surgery. However, reported rates of wound complications in the literature vary widely, and the underlying risk factors remain insufficiently understood. This study shows that larger tumors, tumors involving the urethra, or located near the urethra or the perineum are at higher risk of especially wound breakdowns. Primary skin closure can be challenging due to tension and anatomical disruption, often requiring reconstructive surgery to restore form and function. Although reconstruction is associated with higher wound complication rates and longer hospital stays—likely due to the complexity of these selected cases—it can improve quality of life for selected patients. Reconstructive surgery is best reserved for large tumors, urethral involvement, or tumors located near the urethra or on the perineum. In contrast, small tumors suitable for primary closure may not benefit from reconstructive surgery. Multidisciplinary planning is essential to indicate the use of reconstructive surgery.

## 1. Introduction

Vulvar cancers are relatively uncommon, with a higher and increasing incidence in Europe and North America. Worldwide, up to 42,240 women were diagnosed with vulvar cancer in 2020 [1,2]. In the Netherlands, 456 new cases of vulvar cancer were reported in 2023 [3]. The vast majority of vulvar cancers are squamous cell carcinoma (VSCC) (70–90%), and the minority are basal cell carcinoma, adenocarcinoma, and melanoma. Vulvar cancer usually presents as a persistent vulvar lesion, ulceration, itching, or pain [4]. Over the last few years, there has been a striking increase in incidence, mainly in women aged <60 years [5]. VSCC has a 5-year survival rate of 75%, which varies significantly by stage, from 84% in FIGO stage I to 35% in FIGO stage IV [6].

VSCC development distinguishes two pathophysiological pathways; the human papillomavirus positive pathway (HPV+), which accounts for approximately 20% of all vulvar cancers, and HPV-negative tumors, which account for the remaining 80% [7]. HPV-positive tumors have the most favorable outcomes in terms of overall survival, relative survival (RS), and recurrence-free period (RFD) [8]. The HPV-negative pathway is caused by dysplastic changes in the vulvar epithelium associated with lichen sclerosis (LS) [9]. Differentiated vulvar intraepithelial neoplasia (dVIN) is the main precursor for HPV-negative VSCC [10]. Recently, within the HPV-negative pathway, a distinct vulvar cancer cohort is identified by subclassification upon mutations in the p53 gene [8]. The HPV-negative/p53 mutant cohort, accounting for 15% of VSCC, has the worst survival and tends to recur more often compared to the HPV-negative/p53 wild-type VSCC cohort, accounting for 66% of vulvar cancers in this study [8].

Despite different pathophysiological pathways of VSCC the treatment of vulvar cancer at present is similar and consists of a surgical resection in the majority of patients or chemo- and/or radiotherapy in case of more advanced stages [10]. Vulvar surgery aims to radically remove the vulvar cancer lesion with clear margins and minimize the effect on the surrounding tissue and functional anatomical critical structures [11,12,13]. In surgical treatment for early stage VSCC a wide local tumor resection (WLE) is in general combined with either groin surgery by sentinel node (SN) procedure or inguinofemoral lymphadenectomy (IFL) [7,8,9,10].

Vulvar surgery is often experienced as mutilating by anatomical distortion and has a severe negative impact on functional, psychological, and sexual functioning [14]. Surgical complications are common in vulvar cancer surgery and increase with the extent and level of radicality of surgery [15]. Wound complication rates are reported in a wide range of 9% to 58% of patients following vulvectomy [16,17,18]. Risk factors for these wound complications are less well-known and are associated with tumor diameter and combination with groin surgery [15]. Radiotherapy is known to impair wound healing, necessitating careful consideration of this factor when planning (reconstructive) surgery [19].

Depending on the size and location of the tumor, primary skin closure may cause severe tension and difficulty in preserving anatomy and function, necessitating reconstructive surgery with skin transposition to restore the external genitalia post-surgery. Reconstructive surgery may therefore aid in improved quality of life [20]. Different reconstructive techniques in the vulvar area are described as the lotus petal flap, VY-plasty, gracilis or anterolateral thigh (ALT) flap [21]. Research shows that reconstructions with flaps yield more favorable results for perineal tumors than primary closure [22,23,24,25]. A reconstructive technique for vulvar cancer can either be performed by the gynecological oncologist and/or a consulting reconstructive surgeon [3,26].

In this study, we aim to determine the incidence and risk factors for wound complications after vulvar cancer surgery. As a secondary aim, we compare the effects of primary closure versus reconstructive surgery on wound complications.

## 2. Materials and Methods

### 2.1. Patients

This retrospective multi-center cohort study was conducted in four tertiary referral centers for vulvar cancer in the Netherlands: Catharina Hospital Eindhoven, Amsterdam University Medical Centers (location AMC), Maastricht University Medical Center, and Radboud University Medical Center Nijmegen. Eligibility criteria were all women diagnosed with a (suspected) primary or recurrent vulvar carcinoma surgically treated in the Catharina Hospital Eindhoven between 2018 and 2021 or in one of the other three between 2018 and 2019. Further inclusion criteria were a minimum age of 18 and surgery planned as treatment in a curative setting. A total of nineteen patients with differentiated Vulvar Intraepithelial Neoplasia (dVIN) have been included because they were suspected of having vulvar cancer preoperatively. Exclusion criteria were previous radiation therapy on the vulva or patients lost to follow-up in the first 6 weeks of follow-up.

### 2.2. Data Handling and Collection

This study was exempted from formal ethical assessment, as stated by the Medical Research Involving Human Subjects Act (WMO). The study followed the Declaration of Helsinki and the Good Clinical Practice guidelines. Institutional approval for this study was obtained from each of the participating centers. Patients’ privacy was protected using anonymized data and maintaining confidentiality throughout the study. Data collection and management were performed using a secure electronic data capture system (Castor eCRF) hosted on a dedicated server. All data were entered directly into the eCRF, and access was restricted to authorized personnel. Collected data included patient and tumor characteristics, operation details, and postoperative variables.

### 2.3. Outcome Measures

The primary outcome measure is the incidence and risk factors for wound complications after vulvar cancer surgery. As a secondary aim, we compare the effects of PC versus RS on wound complications. Wound complications were scored as either one or not multiple (e.g., only breakdown or infection). Variables that were taken into account as possible risk factors included patient characteristics such as age during the procedure, body mass index (BMI), smoking status, comorbidities, and the use of corticosteroids, immunosuppressants, or anticoagulants. Tumor-related factors considered were localization of the tumor, tumor diameter, whether the tumor was primary or recurrent, and proximity to the urethra, clitoris, or midline. Surgical factors included the type of surgical therapy, the closure method of the vulva (e.g., primary closure vs. reconstruction), the use of pre-operative antibiotics, the suture technique and material used, the use of clitoridectomy, and whether resection of the urethra was performed. Postoperative factors, such as the total duration of drainage after surgery, number of days until mobilization, and the sitting schedule during hospitalization, were also taken into account.

### 2.4. Complication Definitions

Wound complications were categorized as follows: vulvar wound complications occurring during hospitalization or within six weeks postoperatively, including wound breakdown, wound infection, and severe hematoma. Since the literature lacks a definition of wound complications after vulvar surgery, a meeting with four gynecologic oncologists and two reconstructive surgeons was performed. It was determined that if a wound breakdown was mentioned in the medical record, it was documented as such. Wound breakdown was further categorized into three classes. Breakdown of less than 25% of the resection was defined as mild wound breakdown. Breakdown between 25% and 50% was considered moderate. Breakdown exceeding 50% was classified as severe. Wound infections were defined as skin infections requiring antibiotics or surgical debridement. If clinical photographs were available, they were used for the assessment; however, in cases where photographs were not present, we relied on written documentation in the medical records.

### 2.5. Definition of Tumor-Free Margin

Tumor-free margins were defined as margins in which the pathology report indicated that a surgical margin was tumor-free.

### 2.6. Data Analysis

Normality was tested using the Kolmogorov–Smirnov test. Continuous variables were summarized using either means and standard deviations or medians and interquartile ranges (IQRs), depending on the distributional characteristics of the data. Categorical variables were summarized using frequencies and numbers. Analyses and comparisons were performed between primary and reconstructive closure. Numeric data were analyzed using the Student *t*-test or Mann–Whitney test depending on normality. Categorical data were analyzed using the chi-squared test or Fisher’s Exact test in the case of small numbers. Risk factors for vulvar wound complications were assessed using univariate and multivariate logistic regression analyses. In the univariate logistic regression, we evaluated potential risk factors individually to identify those with a significant association (*p* < 0.10) with wound complications. This more lenient threshold was applied to avoid the premature exclusion of potentially relevant variables or confounds. Significant factors identified in the univariate analysis were subsequently included in the multivariate logistic regression analysis. This allows for assessing the independent effects of these factors on wound complications while adjusting for other variables. Subgroup analyses were performed for different tumor sizes; <2 cm, 2–4 cm and >4 cm. The data was analyzed using SPSS version 29 (Statistical Package for the Social Sciences).

## 3. Results

This study included 394 women surgically treated for vulvar carcinoma during the study period. Overall, patients had a mean age of 69 years, and 20.1% of all patients smoked. Histology of vulvar cancer diagnosis is squamous cell carcinomas in 91.1%, 3.0% melanoma, 4.8% dVIN, and 1.0% adenocarcinoma. Of the included patients, 324 (82.2%) had a primary tumor, and 70 (17.8%) had a recurrent tumor. The mean tumor size was 2.7 cm, and 64.0% of the tumors were located within 1 cm of the midline, with 31.7% of the tumors located anterior (clitoral) and 9.9% of the tumors located posterior (perineal). In 39.3% of the patients, a clitorectomy was required, and in 20.1%, part of the urethra was removed during surgery (Table 1).

### 3.1. Wound Complications After Vulvar Cancer Surgery

**Figure 1:**
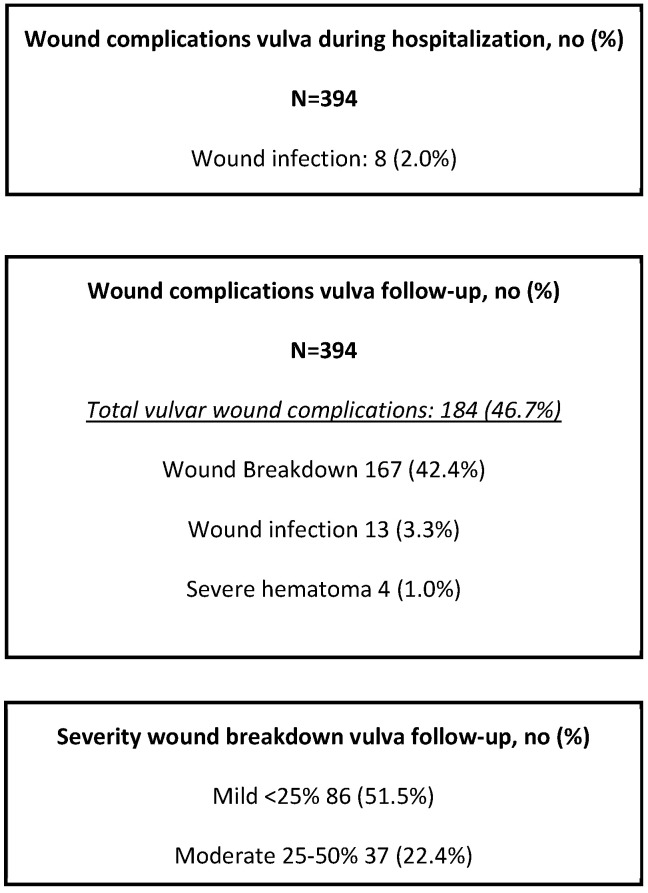
Wound complications after original vulvar surgery during hospitalization and follow-up.

During follow-up, vulvar wound complications were reported in 184 patients (46.7%). Three patients developed wound infections (3.3%), and 167 developed wound breakdowns (42.4%). Of the wound breakdown, 86 of these were mild (51.5%), 37 were moderate (22.4%), and 44 were severe (26.7%) (Figure 1).

#### Risk Factors for Wound Complications

A comparison of baseline characteristics and tumor characteristics between the groups with and without wound complications is presented in Table 2. Factors associated with an increased likelihood of wound complications include larger tumor size, with a mean diameter of 3.0 cm in the group with wound complications compared to a mean tumor diameter of 2.4 cm in the group without wound complications (*p* = 0.001). Furthermore, proximity to the urethra (*p* = 0.035), resection of the urethra during surgery (*p* = 0.041), and a perineal tumor location (*p* = 0.003) are associated with an increased risk of wound complications (Table 2). However, in our multivariate analyses, no significant odds ratios were found (Table 3).

### 3.2. Reconstructive Surgery in Vulvar Cancer Surgery

Reconstructive surgery included various procedures, such as VY-plasty (*n* = 60), Lotus Petal flaps (*n* = 12), Gracilis or ALT flaps (*n* = 2), and posterior vaginal wall plasty (*n* = 2). Table 4 includes an overview listing all the characteristics that were compared between the reconstructive surgery and the primary closure group. The group of patients operated on with a reconstructive method (*n* = 76) significantly included more recurrent tumors: 26.3%, compared to 15.7% in the primary closure group (*n* = 318) (*p* = 0.030). Reconstructive surgery was most often used in patients with tumors bigger than 4 cm (*p* < 0.001), tumors located within 1 cm of the midline (*p* = 0.013), within 1 cm of the anus (*p* < 0.001) and 1 cm of the urethra (*p* < 0.001).

#### 3.2.1. Impact of Reconstructive Surgery on Wound Complications

The group that had reconstructive surgery (*n* = 76) had significantly more complications than primary closure: 55 (72.4%) vs. 129 (40.6%) (*n* = 318) (*p* < 0.001) (Table 4). However, the indications for reconstructive surgery included tumors larger than 4cm, perineal-located tumors, and recurrent lesions, which may contribute to the increased complication rate. Wound breakdowns are reported to be significantly less frequent in patients after primary closure, 34.6% (*n* = 110), compared to after reconstructive surgery, 69.7% (*n* = 53). Remarkably, the group of patients undergoing reconstructive surgery had significantly fewer wound breakdowns in the category of severe wound breakdowns (those that exceed 50%): 21.8% compared to 28.6% for the primary closure group (*p* = 0.03). The multivariate logistic regression analysis revealed that the group with reconstructive surgery had an increased risk of wound complications OR 1.1 (95% confidence interval [CI]: 1.1–1.2) compared to the primary closure group (Table 4).

#### 3.2.2. Wound Breakdown Categorized by Size Classification

The incidence of wound breakdowns in smaller tumors (<2 cm) was 39.8%. The reconstructive surgery group showed more wound breakdowns than the primary closure group (63.2% versus 36.7%) (*p* = 0.027). In 35.3% of reconstructive cases < 2 cm, the tumor was a recurrent tumor. Having previously undergone excision of the primary tumor, the availability of local tissue for closure is significantly reduced at the affected site. For the tumor size group between 2 and 4 cm, wound breakdown incidence was 50.0%. Breakdowns were comparable for primary closure and reconstructive surgery: 47.4% versus 66.7% (*p* = 0.129). The total incidence of wound breakdowns for more extensive tumors (>4 cm) was 54.3%. In the categorized analyses, a tumor size greater than 4.0 cm in diameter was identified as a risk factor in the univariate analysis (OR 1.9; 95% CI 1.1–3.1) (Table 3). The reconstructive surgery group experienced more wound breakdowns (79.5% versus 36.4%) (*p* ≤ 0.001). (Table 5).

#### 3.2.3. Impact of Reconstructive Surgery on Resection Margins

In the reconstructive surgery group, 76.3% of patients achieved free tumor margins, with 85.8% achieving comparable results (*p* = 0.042) in the primary closure group. In the different tumor size groups, the percentage of achieved tumor-free margins decreased gradually with the extension of tumor sizes irrespective of primary or reconstructive closure (Table 6).

### 3.3. Hospitalization and Follow-Up Variables After Vulvar Cancer Surgery

Patients of the reconstructive method group were significantly longer hospitalized with a median of 5 (IQR 3–7) days versus a median of 2 days (IQR 1–4) (*p* < 0.001; Table 7). Their catheter stayed in longer as well (4 days versus 1 day (*p* < 0.001)). Re-hospitalization occurred in 52 patients (13.2%); with similar numbers in the reconstructive and the primary group (11 patients (14.5%) versus 41 patients (12.9%)) (*p* = 0.833). However, patients in the reconstructive closure group were re-hospitalized longer; 8 days (3–22) versus 3 days (1–6) (*p* = 0.055).

## 4. Discussion

In this retrospective multicenter study with a large cohort of 394 patients with vulvar cancer, we report a total of 46.7% of wound complications after surgery for vulvar cancer. Large tumor size, proximity to the urethral, urethra resection, and perineal tumor location were identified as potential contributors to wound complications. In multivariate analysis, reconstructive surgery is associated with a higher risk of wound breakdown. Albeit, this may reflect selection bias, as reconstructive surgery is often preemptively chosen for cases involving larger tumors > 4 cm, perineal-located tumors, and recurrent lesions, which inherently carry higher risks of wound breakdown. Notably, in reconstructive surgery, the wound breakdown tends to be less severe.

The wide variation in reported rates of wound complications in vulvar surgery (9–58%) suggests that there is still some uncertainty regarding their prevalence. According to several articles, tumor size appears to be an essential risk factor for wound complications [15,16,17]. Boyles et al. reported a 42.7% incidence of wound complications in vulvar resections for non-malignant cases, with 39.6% experiencing wound breakdowns and 6.5% developing infections. Risk factors identified for wound complications were larger tumors (OR 1.03; 95% CI 1.01–1.05) and perineal located tumors (OR 2.25; 95% CI 1.38–3.66), which aligns with our findings.

We evaluated several variables as potential risk factors, e.g., smoking, type of suture technique or suture material, mobilization protocols, use of urinary catheter, and others (Appendix A Table A1). The risk factors we identified were larger tumor diameter > 4 cm (OR 1.9; 95% CI 1.1–3.1), <1 cm distance to the anus (OR 3.1; 95% CI 1.7–5.7), resection of the urethra (OR 1.7; 95% CI 1.0–2.8), and perineal location of the tumor (OR 3.2; 95% CI 1.5–6.8). However, after adjusting for confounds, we had no significant risk factors besides RS (OR 1.1; 95% CI 1.1–1.2). Our data indicate that there is no difference in the timing of catheter removal. These risk factors mark important clinical parameters physicians should consider in preparation for vulvar surgery and patient counseling.

The use of reconstructive surgery in vulvar cancer surgery may be beneficial in surgical outcomes for patients with vulvar carcinomas. Panici et al. demonstrated significantly improved outcomes after reconstructive surgery in tumors > 4 cm, with an 11% incidence of wound breakdowns following VY-flaps compared to 40% wound breakdowns with primary closure [27]. In our study, however, we only found wound breakdowns to be less severe after reconstructive surgery. Previous research shows that reconstructive surgery improves clinical outcomes by restoring the anatomy and function of the external genitalia. Reconstructive surgery may therefore aid in improved quality of life [22,23].

Reconstructive surgery is now most often indicated and used in tumors with a larger diameter. In our data set, the median tumor diameter in patients in the reconstructive surgery group is 4.0 cm (range 4.0–5.0 cm) compared to 2.0 cm (range 2.0–2.5 cm) in the primary closure group. Aviki et al. [28] reported similar numbers with an average size of 3.73 cm vs. 2.03 cm, and reconstructive surgery was used more in recurrent cancers or after previous radiotherapy without impact on wound complications. Only previous radiotherapy was associated with wound complications (OR 17) in this study. In our study cohort, previous radiotherapy was an exclusion criterion based on these study data, and we still report high numbers of wound healing disorders. In other studies, however, as published by Weikel et al. [29], no effects in wound healing after radiotherapy were reported as reconstructive surgery was used.

In the Netherlands, reconstructive surgery for vulvar cancer follows local protocols, as the Dutch guidelines provide no specific recommendations for when to use reconstructive surgery [30]. The European Society of Gynaecological Oncology advises including reconstructive skills in multidisciplinary teams. At the same time, the British Gynaecological Cancer Society recommends it for posterior and larger lateral lesions to aid closure and preserve vaginal function [31,32]. Some reports provide algorithms for vulvar reconstruction techniques but need more recommendations on their indications [33]. Multidisciplinary collaboration is vital for providing high-quality care. Considering our data, reconstruction should be considered in larger tumor size, proximity to the urethra, resection of the urethra during surgery, and perineal tumor location. The safety margin recommendations in vulvar cancer vary from margins > 3 mm to those > 8 mm [34,35]. Reconstructive surgery has been reported to facilitate higher complete resection rates [36]. However, our results show that most patients had tumor-free margins, with a significant difference favoring the primary closure group over the reconstructive group.

Limitations of our study are the retrospective nature of the study, the lack of definitions of wound complications used, and the difference in physical follow-up visits in different centers. The lack of standardized wound assessment forms across centers makes for variability in physical follow-up visits, potentially influencing the reported complication rates. The absence of universal definitions for wound complications has been recognized as a recurring challenge, as highlighted in the literature [37]. We have chosen to use the cut-off points according to the percentage of the wound affected. However, in many cases, it proved difficult to place the retrospective data in one of these categories. Muallem et al. previously chose to evaluate wound complications using the Clavien Dindo system. It is a scale that only sometimes reflects the differences and nuances, which is why we have not opted for this classification [38].

In our dataset a selection bias to consult a reconstructive surgeon is present since the gynecologist selects patients where closure on primary intent is impossible based on previous experience. This is important in interpreting the results that show that the use of reconstructive surgery increases the risk of wound complications. The reconstructive surgeons in general are consulted in case of larger tumors, perineal located tumors, and recurrent lesions. These factors are independent of the surgical technique but can influence wound complications. We believe that selection bias does not negatively impact the generalizability of our results. Selection bias is also present in clinical practice, and as such, it provides a good reflection of real-world clinical scenarios.

Despite these limitations, our results provide valuable insights into the high prevalence of wound complications, reflecting current clinical practice in four gynecologic oncology centers in the Netherlands.

Further research is needed to identify effective strategies for the prevention of wound complications. There is some preventive evidence for vacuum-assisted therapy with Prevena/PICO plasters [39]. However, in the urogenital area, application is complex, and evidence in this anatomical area is lacking. To improve outcomes of vulvar cancer surgery, future studies should focus on healthcare evaluation to prospectively evaluate the current practice and the impact of (reconstructive) surgical treatment on health-related patient quality of life, including daily functioning, sexual functioning, and body image.

## 5. Conclusions

In conclusion, larger tumor size, tumor proximity to the urethra, resection of the urethra during surgery, and a perineal tumor location are associated with an increased risk of wound complications after vulvar cancer surgery. RS is associated with high wound complication rates in vulvar cancer and is associated with more extended hospitalization, though this is related to case selection. Based on these data, RS should not be advised in small tumors where primary closure is possible. The indications of reconstructive surgery in vulvar cancer based on these data should be large tumors, when the urethra has to be resected during surgery, or when the tumor is located in proximity to the urethra or perineum. Multidisciplinary collaboration in vulvar cancer surgery is essential to indicate the use of reconstructive surgery.

## Figures and Tables

**Table 1:** Baseline patient characteristics.

Overall *n* = 394		Primary/Recurrent	
**Age in years**, mean (SD)	69 (14)	Primary tumor	324 (82.2%)
		Recurrent tumor	70 (17.8%)
**BMI kg/m^2^**			
<20	17 (4.3%)	**Localization tumor**	
20–25	138 (35.0%)	<1 cm midline	252 (64.0%)
25–30	118 (29.9%)	>1 cm midline	142 (36.0%)
>30	121 (30.7%)		
		**Proximity to anus**	
**Smoking**	77 (20.1%)	<1 cm	57 (14.5%)
		1–2 cm	34 (8.6%)
**Comorbidities**		>2 cm	303 (76.9%)
DM (type I and II)	56 (14.2%)		
Lichen Sclerosis	123 (31.2%)	**Proximity to urethra**	
Heart- and vascular disease	215 (54.6%)	<1 cm	106 (26.9%)
		1–2 cm	129 (32.7%)
**Medication**		>2 cm	159 (40.4%)
Corticosteroids	130 (33.0%)		
Anti-coagulants	104 (26.4%)	**Surgical therapy**	
Immunosuppressive	11 (2.8%)	WLE + SN	201 (51.0%)
		WLE + LAD and/or debulking	110 (27.9%)
**Location Tumor**		WLE, no groins	83 (21.1%)
Clitoris	125 (31.7%)		
Perineum	39 (9.9%)	**Type of closure**	
Labium minora	105 (26.7%)	Primary closure	318 (80.7%)
Labium majora	48 (12.1%)	Alternative closure	76 (19.3%)
Labium majora + minora	43 (10.9%)		
		**Clitorectomy**	155 (39.3%)
**Diameter of the tumor (clinical)**		**Resection of urethra**	79 (20.1%)
Diameter in cm, median (95% CI)	2.5 (2.5–3.0)		
Categorical in cm		**Diagnosis**	
<2 cm	166 (42.1%)	PCC	359 (91.1%)
2–4 cm	134 (34.0%)	Melanoma	12 (3.0%)
>4 cm	94 (23.9%)	dVIN	19 (4.8%)
		Adenocarcinoma (incl. M. Paget)	4 (1.0%)

Data are presented as mean (standard deviation), number (percentage), or median (interquartile range). BMI, body mass index; DM, diabetes mellitus; WLE, wide local excision; SN, sentinel node; LAD, lymphadenectomy; PCC, squamous cell carcinoma.

**Table 2:** Comparison of patient and tumor characteristics: wound complications vs. no complications.

	Overall *n* = 394	Wound Complications *n* = 184	No Complications *n* = 210	*p*-Value
Age in years, mean (SD)	69 (14)	70 (13)	68 (15)	0.388
**BMI kg/m^2^**				0.693
<20, no (%)	17 (4.3%)	8 (4.3%)	9 (4.3%)	
20–25, no (%)	138 (35.0%)	59 (32.1%)	79 (37.6%)	
25–30, no (%)	118 (29.9%)	59 (32.1%)	59 (28.1%)	
>30, no (%)	121 (30.7%)	58 (31.5%)	63 (30.0%)	
Smoking, no (%)	77 (20.1%)	42 (23.3%)	35 (17.2%)	0.240
**Comorbidities**				
DM (type I and II), no (%)	56 (14.2%)	21 (11.4%)	35 (16.7%)	0.136
Lichen Sclerosis, no (%)	123 (31.2%)	51 (27.7%)	72 (34.3%)	0.160
Heart- and vascular disease, no (%)	215 (54.6%)	107 (58.2%)	108 (51.4%)	0.181
**Medication**				
Corticosteroids, no (%)	130 (33.0%)	59 (32.1%)	71 (33.8%)	0.807
Anti-coagulants, no (%)	104 (26.4%)	51 (27.7%)	53 (25.2%)	0.578
Immunosuppressive, no (%)	11 (2.8%)	4 (2.2%)	7 (3.3%)	0.486
**Preoperative antibiotics**				
Yes, no (%)	355 (90.1%)	171 (92.9%)	184 (87.6%)	0.130
No, no (%)	39 (9.9%)	13 (7.1%)	26 (12.4%)	
**Location Tumor**				
Clitoris, no (%)	125 (31.7%)	52 (28.3%)	73 (34.8%)	0.167
Perineum, no (%)	39 (9.9%)	27 (14.7%)	12 (5.7%)	0.003
Labium minora, no (%)	105 (26.7%)	44 (23.9%)	61 (29.0%)	0.250
Labium majora, no (%)	48 (12.1%)	24 (13.0%)	24 (11.4%)	0.625
**Diameter of the tumor**				0.001
Diameter in cm, mean (SD)	2.7 (1.7)	3.0 (1.8)	2.4 (1.5)	
Categorical in cm, no (%)				0.051
1–2 cm, no (%)	166 (42.1%)	66 (35.9%)	100 (47.6%)	
2–4 cm, no (%)	134 (34.0%)	67 (36.4%)	67 (31.9%)	
>4 cm, no (%)	94 (23.9%)	51 (27.7%)	43 (20.5%)	
**Primary/recurrent**				0.330
Primary tumor, no (%)	324 (82.2%)	155 (84.2%)	169 (80.5%)	
Recurrent tumor, no (%)	70 (17.8%)	29 (15.8%)	41 (19.5%)	
**Localization tumor**				0.438
<1 cm_midline, no (%)	252 (64.0%)	114 (62.0%)	138 (65.7%)	
>1 cm_midline, no (%)	142 (36.0%)	70 (38.0%)	72 (34.3%)	
**Proximity to anus**				<0.001
<1 cm, no (%)	57 (14.5%)	39 (21.2%)	18 (8.6%)	
1–2 cm, no (%)	34 (8.6%)	21 (11.4%)	13 (6.2%)	
>2 cm, no (%)	303 (76.9%)	124 (67.4%)	179 (85.2%)	
**Surgical therapy**				0.727
WLE, no groins, no (%)	110 (27.9%)	53 (28.8%)	57 (27.1%)	
WLE + SN, no (%)	201 (51.0%)	90 (48.9%)	111 (52.9%)	
WLE + LAD and/or debulking, no (%)	83 (21.1%)	41 (22.3%)	42 (20.0%)	
**Type of closure**				<0.001
Primary closure, no (%)	318 (80.7%)	129 (70.1%)	189 (90.0%)	
Alternative closure, no (%)	76 (19.3%)	55 (29.9%)	21 (10.0%)	
**Operation variables vulva**				
Clitorectomy, no (%)	155 (39.3%)	68 (37.0%)	87 (41.4%)	0.365
Resection of urethra, no (%)	79 (20.1%)	45 (24.5%)	34 (16.2%)	0.041
**Diagnosis**				0.777
PCC, no (%)	359 (91.1%)	170 (92.4%)	189 (90.0%)	
Melanoma, no (%)	12 (3.0%)	5 (2.7%)	7 (3.3%)	
Adenocarcinoma (incl. M. Paget), no (%)	4 (1.0%)	1 (0.5%)	3 (1.4%)	
dVIN	19 (4.8%)	8 (4.3%)	11 (5.2%)	

Data are presented as mean (standard deviation) or number (percentage). BMI, body mass index; DM, diabetes mellitus; WLE, wide local excision; SN, sentinel node; LAD, lymphadenectomy; PCC, squamous cell carcinoma; WLE, wide local excision; PCC, squamous cell carcinoma.

**Table 3:** Univariate logistic regression and multivariate logistic regression analysis for wound complications in patients with vulvar cancer.

	Univariate Logistic Regression		Multivariate Logistic Regression	
	OR, 95% CI	*p*-Value	OR, 95% CI	*p*-Value
Age in years	1.006 (0.992–1.020)	0.388	-	-
Localization tumor to midline	1.177 (0.779–1.777)	0.438	-	-
Diameter of the tumor in cm	1.226 (1.082–1.389)	0.001	-	-
Tumor category < 2 cm	1.000			
Tumor category 2–4 cm	1.269 (0.797–2.021)	0.315	1.240 (0.759–2.027)	0.390
Tumor category > 4 cm	1.892 (1.141–3.137)	**0.013**	1.257 (0.710–2.227)	0.433
Does smoke	1.000			
Does not smoke	1.546 (0.926–2.581)	0.096	1.413 (0.811–2.461)	0.222
Stopped smoking	1.248 (0.718–2.169)	0.432	1.388 (0.770–2.500)	0.276
BMI < 20 kg/m^2^	1.190 (0.433–3.269)	0.736	-	-
BMI 20–25 kg/m^2^	1.000			
BMI 25–30 kg/m^2^	1.339 (0.817–2.194)	0.247	-	-
BMI > 30 kg/m^2^	1.233 (0.755–2.014)	0.403	-	-
Tumor located around the clitoris	1.000			
Perineal tumor location	3.159 (1.466–6.804)	**0.003**	1.668 (0.597–4.664)	0.329
Tumor located on the left Labium minora	1.106 (0.592–2.066)	0.752	1.079 (0.543–2.145)	0.828
Tumor located on the right Labium minora	0.902 (0.452–1.801)	0.771	0.854 (0.399–1.825)	0.683
Tumor located on the left Labium majora	1.531 (0.628–3.737)	0.349	1.754 (0.668–4.607)	0.254
Tumor located on the right Labium majora	1.296 (0.548–3.067)	0.555	1.407 (0.537–3.684)	0.487
Tumor located on the left Labium minora + majora	1.659 (0.689–3.993)	0.259	1.436 (0.549–3.756)	0.461
Tumor located on the right Labium minora + majora	1.021 (0.384–2.714)	0.967	1.092 (0.381–3.130)	0.870
Tumor located around the introitus	1.248 (0.583–2.672)	0.569	0.935 (0.399–2.192)	0.877
Clitorectomy	0.829 (0.552–1.244)	0.365	-	-
Proximity to Clitoris < 1 cm	1.000			
Proximity to Clitoris 1–2 cm	1.261 (0.749–2.123)	0.384	1.223 (0.697–2.145)	0.483
Proximity to Clitoris > 2 cm	1.543 (0.980–2.429)	**0.061**	1.066 (0.592–1.922)	0.831
Proximity to Anus < 1 cm	3.128 (1.710–5.720)	**<0.001**	1.829 (0.861–3.884)	0.116
Proximity to Anus 1–2 cm	2.332 (1.125–4.832)	**0.023**	1.680 (0.747–3.778)	0.209
Proximity to Anus > 2 cm	1.000			
Resection of urethra	1.676 (1.019–2.757)	**0.042**	1.458 (0.838–2.537)	0.182
Reconstructive surgery	1.183 (1.104–1.267)	**<0.001**	**1.134 (1.050–1.225)**	**<0.001**

Data are presented as ODDS RATIO and 95% CI intervals. Every variable with *p* < 0.1 was used in the multivariate. Significance < 0.05; are highlighted in bold.

**Table 4:** Comparison of primary closure and reconstructive surgery.

	Overall *n* = 394	Primary Closure *n* = 318	Reconstructive Method *n* = 76	*p*-Value
**Primary/recurrent**				0.030
Primary tumor, no (%)	324 (82.2%)	268 (84.3%)	56 (73.7%)	
Recurrent tumor, no (%)	70 (17.8%)	50 (15.7%)	20 (26.3%)	
**Diameter of the tumor**				
Diameter in cm, median (IQR)	2.50 (2.8)	2.00 (2.0)	4.00 (3.0)	0.004
Categorical in cm, no (%)				<0.001
1–2 cm, no (%)	166 (42.1%)	147 (46.2%)	19 (25.0%)	
2–4 cm, no (%)	134 (34.0%)	116 (36.5%)	18 (23.7%)	
>4 cm, no (%)	94 (23.9%)	55 (17.3%)	39 (51.3%)	
**Localization tumor**				0.013
<1 cm midline, no (%)	252 (64.0%)	194 (61.0%)	58 (76.3%)	
>1 cm midline, no (%)	142 (36.0%)	124 (39.0%)	18 (23.7%)	
**Proximity to anus**				<0.001
<1 cm	57 (14.5%)	29 (9.1%)	28 (36.8%)	
1–2 cm	34 (8.6%)	22 (6.9%)	12 (15.8%)	
>2 cm	303 (76.9%)	267 (84.0%)	36 (47.4%)	
**Proximity to urethra**				<0.001
<1 cm	106 (26.9%)	75 (23.6%)	31 (40.8%)	
1–2 cm	129 (32.7%)	116 (36.5%)	13 (17.1%)	
>2 cm	159 (40.4%)	127 (39.9%)	32 (42.1%)	
**Operation variables vulva**				
Clitorectomy, no (%)	155 (39.3%)	125 (39.3%)	30 (39.5%)	0.979
Resection of urethra, no (%)	79 (20.1%)	54 (17.0%)	25 (32.9%)	0.002
Perineal no (%)	39 (9.9%)	20 (6.3%)	19 (25.0%)	<0.001
**Wound complications**				
Total vulvar wound complications, no (%)	184 (46.7%)	129 (40.6%)	55 (72.4%)	<0.001
Wound breakdowns, no (%)	167 (42.4%)	110 (34.6%)	53 (69.7%)	
Wound infection, no (%)	13 (3.3%)	14 (4.4%)	2 (2.6%)	
Severity wound breakdowns				0.026
Mild < 25%, no (%)	86 (51.5%)	62 (55.4%)	24 (43.6%)	
Moderate 25–50%, no (%)	37 (22.2%)	18 (16.1%)	19 (34.5%)	
Severe > 50%, no (%)	44 (26.7%)	32 (28.6%)	12 (21.8%)	

Data are presented as mean (standard deviation), number (percentage), or median (interquartile range). Numeric data were analyzed using the Student *t*-test and Mann-Whitney U test. Categorical data were analyzed using the chi-squared test or Fisher’s Exact Test.

**Table 5:** Comparison of reconstructive and primary closure per tumor size complications.

	Overall *n* = 394	Primary Closure *n* = 318	Reconstructive Method *n* = 76	*p*-Value
**Tumors < 2 cm** *(n = 166)*		*(n = 147)*	*(n = 19)*	
Wound complications until 6 weeks	66/166 (39.8%)	54/147 (36.7%)	12/19 (63.2%)	0.027
**Tumors 2–4 cm** *(n = 134)*		*(n = 116)*	*(n = 18)*	
Wound complications until 6 weeks	67/134 (50.0%)	55/116 (47.4%)	12/18 (66.7%)	0.129
**Tumors > 4 cm** *(n = 94)*		*(n = 55)*	*(n = 39)*	
Wound complications until 6 weeks	51/94 (54.3%)	20/55 (36.4%)	31/39 (79.5%)	<0.001

Data are presented as numbers (percentage). Categorical data were analyzed using the chi-squared test.

**Table 6:** Comparison of reconstructive and primary closure per tumor size—free margins.

	Overall *n* = 394	Primary Closure *n* = 318	Reconstructive Method *n* = 76	*p*-Value
**Total group**				
Tumor margin free	331 (84.0%)	273 (85.8%)	58 (76.3%)	0.042
**Tumors < 2 cm** *(n = 166)*		*(n = 147)*	*(n = 19)*	
Tumor margin free	150 (90.4%)	132 (89.8%)	18 (94.7%)	0.492
**Tumors 2–4 cm** *(n = 134)*		*(n = 116)*	*(n = 18)*	
Tumor margin free	114 (85.1%)	100 (86.2%)	14 (77.8%)	0.350
**Tumors > 4 cm** *(n = 94)*		*(n = 55)*	*(n = 39)*	
Tumor margin free	67 (71.3%)	41 (74.5%)	26 (66.7%)	0.406

Data are presented as number (percentage). Categorical data were analyzed using the chi-squared test or Fisher’s Exact Test.

**Table 7:** Hospitalization variables and wound complications after primary vulvar surgery.

	Overall *n* = 394	Primary Closure *n* = 318	Reconstructive Method *n* = 76	*p*-Value
Duration hospitalization, median (IQR)	2 (1–4)	2 (1–4)	5 (3–7)	**<0.001**
Total duration catheter, median (IQR)	1 (1–4)	1 (1–2)	4 (1–7)	**<0.001**
Total duration drains, median (IQR)	6 (5–10)	7 (5–11)	5 (5–9)	0.817
Days till mobilization, median (IQR)	1 (1–1)	1 (1–1)	1 (1–2)	**<0.001**
Re-hospitalization, no (%)	52 (13.2%)	41 (12.9%)	11 (14.5%)	0.833
Duration re-hospitalization, median (IQR)	3 (1–8)	3 (1–6)	8 (3–22)	0.055
Contact with hospital on own request, no (%)	39 (76.5%)	29 (72.5%)	10 (90.9%)	0.202

Data are presented as number (percentage) or median (interquartile range). Categorical data were analyzed using the chi-squared test. Significance < 0.05; are highlighted in bold.

## Data Availability

The raw data supporting the conclusions of this article will be made available by the authors on request.

## Appendix A

**Table A1:** Univariate logistic regression analysis for wound complications in patients with vulvar cancer.

	Univariate Logistic Regression		Multivariate Logistic Regression	
	OR, 95% CI	*p*-Value	OR, 95% CI	*p*-Value
Suture technique vulva	1.065 (0.682–1.661)	0.783	0.495 (0.122–2.007)	0.325
Suture type vulva	2.089 (1.040–4.194)	0.038	0.613 (0.104–3.613)	0.588
Total duration drains	0.967 (0.909–1.029)	0.285	1.009 (0.942–1.081)	0.794
Days till mobilization	0.999 (0.996–1.002)	0.556	0.984 (0.934–1.037)	0.553
Sitting schedule during hospitalization	0.279 (0.126–0.614)	0.002	1.730 (0.330–9.067)	0.517
Reconstructive surgery	1.183 (1.104–1.267)	<0.001	1.299 (1.087–1.551)	0.004

Data are presented as ODDS RATIO, and 95% CI intervals. Every variable was used in the multivariate. Significance < 0.05.
